# Mitochondrial Quality Control Strategies: Potential Therapeutic Targets for Neurodegenerative Diseases?

**DOI:** 10.3389/fnins.2021.746873

**Published:** 2021-11-12

**Authors:** Di Hu, Zunren Liu, Xin Qi

**Affiliations:** ^1^Department of Physiology and Biophysics, Case Western Reserve University School of Medicine, Cleveland, OH, United States; ^2^Department of Biology, College of Arts and Sciences, Case Western Reserve University, Cleveland, OH, United States; ^3^Center for Mitochondrial Disease, Case Western Reserve University School of Medicine, Cleveland, OH, United States

**Keywords:** neurodegenerative diseases, mitochondrial quality control, mitochondrial proteostasis, mitochondrial dynamics, mitophagy

## Abstract

Many lines of evidence have indicated the therapeutic potential of rescuing mitochondrial integrity by targeting specific mitochondrial quality control pathways in neurodegenerative diseases, such as Parkinson’s disease, Huntington’s disease, and Alzheimer’s disease. In addition to ATP synthesis, mitochondria are critical regulators of ROS production, lipid metabolism, calcium buffering, and cell death. The mitochondrial unfolded protein response, mitochondrial dynamics, and mitophagy are the three main quality control mechanisms responsible for maintaining mitochondrial proteostasis and bioenergetics. The proper functioning of these complex processes is necessary to surveil and restore mitochondrial homeostasis and the healthy pool of mitochondria in cells. Mitochondrial dysfunction occurs early and causally in disease pathogenesis. A significant accumulation of mitochondrial damage resulting from compromised quality control pathways leads to the development of neuropathology. Moreover, genetic or pharmaceutical manipulation targeting the mitochondrial quality control mechanisms can sufficiently rescue mitochondrial integrity and ameliorate disease progression. Thus, therapies that can improve mitochondrial quality control have great promise for the treatment of neurodegenerative diseases. In this review, we summarize recent progress in the field that underscores the essential role of impaired mitochondrial quality control pathways in the pathogenesis of neurodegenerative diseases. We also discuss the translational approaches targeting mitochondrial function, with a focus on the restoration of mitochondrial integrity, including mitochondrial dynamics, mitophagy, and mitochondrial proteostasis.

## Introduction

Neurodegenerative disorders (NDs) collectively affect more than 50 million worldwide (Gammon, 2014; GBD 2015 Neurological Disorders Collaborator Group, 2017). The most common NDs include Alzheimer’s disease (AD), Parkinson’s disease (PD), Huntington’s disease (HD), and amyotrophic lateral sclerosis (ALS). Although NDs have been studied for decades, the mechanisms underlying their pathogenesis are still elusive due to the complexity of the disease-causing factors (Bertram and Tanzi, 2005; Jellinger, 2010). Nevertheless, these diseases share some common pathological features: pathological protein aggregation (e.g., Amyloid beta [Aβ] in AD and Lewy bodies in PD) and mitochondrial damage in vulnerable brain regions (Ross and Poirier, 2004; Johri and Beal, 2012). Furthermore, ND-related proteins can directly impair mitochondrial function and trigger cell death. Thus, it is believed that mitochondrial dysfunction plays an important role in the neuronal loss observed with NDs.

Normal mitochondrial function is critical for energy production, calcium buffering, lipid metabolism, and redox regulation that govern cell growth, proliferation, and survival (Antico Arciuch et al., 2012; Giorgi et al., 2018). Sustained ATP production in neurons *via* the electron transport chain (ETC) on the mitochondrial inner membrane (IMM) secures the energy supply for their physiological functions (Mattson et al., 2008). Presynaptic mitochondria serve as a cytosolic calcium reservoir to mediate the release and recycling of neurotransmitters (Billups and Forsythe, 2002; Mattson et al., 2008). Moreover, mitochondria produce and eliminate reactive oxygen species (ROS) during oxidative phosphorylation (OXPHOS). The excessive production of mitochondrial ROS can impair protein function and induce inflammatory responses, leading to neuronal death (Johri and Beal, 2012). Therefore, under stress or disease conditions, quality control mechanisms are required to maintain mitochondrial function. To date, three major mitochondrial quality control (MQC) mechanisms that regulate mitochondrial integrity and maintain mitochondrial functions have been well-characterized: (1) activation of the mitochondrial unfolded protein response (UPR^mt^) can rescue protein homeostasis and bioenergetics (Nargund et al., 2015); (2) mitochondrial dynamics (fission and fusion) maintain mitochondrial morphology and bioenergetics (Chan, 2020; Giacomello et al., 2020); (3) mitophagy pathways eliminate damaged mitochondria *via* various adaptor proteins (e.g., Parkin/PTEN-induced kinase 1 [PINK1]), ensuring a pool of healthy mitochondria (Harper et al., 2018; Pickles et al., 2018).

In most cases of NDs, mitochondrial dysfunction is the result of abnormal MQC. For instance, mitochondrial fragmentation resulting from excessive mitochondrial fission causes bioenergetic deficits in the brain of patients with AD, PD, HD, and ALS (Bueler, 2009; Magrane et al., 2009; Manczak et al., 2011; Shirendeb et al., 2011; Wang W. et al., 2013). Conversely, pharmaceutical manipulations that normalize mitochondrial fission can efficiently rescue mitochondrial morphology and function and increase neuronal survival in various disease models (Guo X. et al., 2013; Filichia et al., 2016; Joshi et al., 2018a,b; Zhao Y. et al., 2019; Hu et al., 2021b). Recent studies have demonstrated that impaired mitophagy is one of the key aspects of the pathogenesis of AD, PD, HD, and ALS (Khalil et al., 2015; Grassi et al., 2018; Fang et al., 2019; Harding et al., 2021). Moreover, the activation of UPR^mt^ has been observed in ND models, and genetic suppression of UPR^mt^ exacerbated the development of neuropathology (Yi et al., 2018; Munoz-Carvajal and Sanhueza, 2020; Zhu et al., 2021). These findings collectively demonstrated the essential role of MQC in keeping neurons healthy and, more importantly, indicate potential therapeutic targets for the treatment of NDs.

In this review, we briefly introduce the current understanding of the molecular basis of MQC, including aspects of the mechanistic pathways, physiological functions, and pathological relevance. We then summarize the current findings of MQC impairment and potential MQC-targeted therapeutic strategies in several NDs. In Tables 1, 2, we summarize the MQC-related proteins and MQC-targeted therapeutic agents, respectively.

**TABLE 1:** Proteins associated with mitochondrial quality control.

Mitochondrial quality control pathway	Protein	Molecular function
Mitochondrial proteostasis	HSP10/60/70/90	Chaperones mediating protein folding
	CLPP/LONP1/AFG3L2/SPG7/YME1L	Mitochondrial proteases mediating degradation
	ATFS1/ATF5/ATF4/FOXO3	Transcription factors stimulating the UPR^mt^ response
	UBL5/DVE1/CHOP/JNK/c-Jun	Co-transcription factors involved in the UPR^mt^ response
	eIF2α/GCN2/PERK	Integrated stress response-related proteins
	PGC1-α/NRF	Transcription factors regulating mitochondrial biogenesis
	SIRT/SOD2	Mitochondrial deacetylases and superoxide dismutase involved in antioxidant regulation and proteostasis
Mitochondrial dynamics	OPA1	IMM GTPase regulating mitochondrial fusion
	MFN1/2	OMM GTPases regulating mitochondrial fusion, transport, and mitophagy
	OMA1/YME1L1	Mitochondrial inner membrane proteases
	DRP1	Cytosolic GTPase regulating mitochondrial division
	MFF/Fis1/MiD49/51	OMM adaptor proteins for DRP1 during mitochondrial division
	Kinesin/dynein	Motor proteins mediating mitochondrial axonal transport
	Miro/TRAK	Adaptor proteins connecting motor proteins to mitochondria
Mitophagy	PINK1	Kinase phosphorylating Parkin
	Parkin	E3-ubiquitin ligase targeting OMM proteins
	VDAC/Mitofusin	OMM proteins
	MPP/PARL	Mitochondrial proteases cleaving PINK1 in healthy mitochondria
	OPTN/TAX1BP1/P62	Autophagy receptor proteins
	VCP/FUNDC1/BNIP3	Mitophagy receptor proteins
	ATG5/ATG7LC3	Proteins mediating autophagy substrate selection and formation of autophagosomes
	UBXN1/UBXD1	UBX domain-containing co-factors
	USP30	Deubiquitinase localized on mitochondria
	GAPDH	Protein mediating glycolysis and serves as a mitophagy adaptor
Others	SIRT3	Mitochondrial deacetylase regulating antioxidants, proteostasis, and mitophagy
	AMPK	Mitochondrial fuel sensor
	LRRK2	PD-associated risk factor; kinase that phosphorylates DRP1 and Miro
	CHCHD10	MICOS subunit regulating cristae structure and mitochondrial contact site
	TOM20	Translocase of the OMM
	TBK1	Kinase regulating apoptosis, autophagy, and inflammation
	PP1	Protein phosphatase dephosphorylating DRP1

**TABLE 2:** Function of therapeutic agents in neurodegenerative diseases.

Disease	Agent	Molecular action	Effects on mitochondria	References
Alzheimer’s disease	Mdivi1	DRP1 inhibitor	Rescues mitochondrial morphology	Xie et al., 2014; Kim et al., 2017; Reddy et al., 2017b, 2018; Yuan et al., 2021
	Szeto-Schiller tetrapeptides 31 (SS31)	Specifically binds to cardiolipin	Rescues mitochondrial morphology; reduces ROS production	Jia et al., 2016; Reddy et al., 2017a
	AP39	H_2_S donor	Rescues mitochondrial morphology; reduces ROS production	Zhao et al., 2016
	Citalopram	Serotonin reuptake inhibitor	Increases bioenergetics	Zhang et al., 2018; Reddy et al., 2021
	TAT-DRP1-SpS	Blocks DRP1 phosphorylation by GSK3β	Rescues mitochondrial morphology	Yan et al., 2015
	DDQ	Inhibits interaction between Aβ and DRP1	Rescues mitochondrial morphology	Kuruva et al., 2017; Vijayan et al., 2021
	S14	Phosphodiesterase (PDE)-7 inhibitor	Regulates mitophagy; rescues mitochondrial morphology	Perez-Gonzalez et al., 2013; Bartolome et al., 2018
	Nicotinamide riboside	NAD + precursor that enhances mitophagy	Enhances clearance of damaged mitochondria	Gong et al., 2013; Xie et al., 2019
	Urolithin A	Metabolite from gut bacteria that enhances mitophagy		Fang et al., 2019; Esselun et al., 2021
	Bexarotene	Promotes mitophagy		Martin-Maestro et al., 2019; Vidal et al., 2021
	Spermidine	Enhances mitophagy		Yang et al., 2020
	Trehalose	Non-reducing disaccharide that enhances mitophagy		Tien et al., 2016
	Resveratrol	Promotes Sirt1-dependent mitophagy		Gu et al., 2021
Parkinson’s disease	P110	DRP1/Fis1 inhibitor	Rescues mitochondrial morphology	Qi et al., 2013; Filichia et al., 2016
	Mdivi1	DRP1 inhibitor	Rescues mitochondrial morphology	Bido et al., 2017
	BC1464	Disrupts the FBXO7/PINK1 interaction	Rescues mitophagy	Liu Y. et al., 2020
	Compound 3	Miro1 reducer	Rescues mitochondrial transport	Hsieh et al., 2019
	Sulfhydration	Hydrogen sulfide donor	Reduces ROS production; Enhance Parkin activity	Vandiver et al., 2013
	Rho-associated protein kinase (ROCK) inhibitors	Rho-associated protein kinase (ROCK) inhibitors	Enhance mitophagy	Moskal et al., 2020
	MitoQ	Augments antioxidant activity of CoQ10	Reduces ROS; rescues mitochondrial morphology	Snow et al., 2010; Solesio et al., 2013; Ojano-Dirain et al., 2014; Xi et al., 2018
	kinetin/kinetin triphosphate (KTP)	ATP analog enhances PINK1 activity	Enhances mitophagy	Hertz et al., 2013; Osgerby et al., 2017
	FT385 and USP30	USP30 inhibitor		Bingol et al., 2014; Rusilowicz-Jones et al., 2020; Luo et al., 2021
	Rapamycin	mTOR activator		Pan et al., 2009; Tain et al., 2009; Crews et al., 2010
Huntington’s disease	Mdivi1	DRP1 inhibitor	Rescues mitochondrial morphology	Manczak and Reddy, 2015; Cherubini et al., 2020
	CHIR99021	Stabilizes calpastatin		Hu et al., 2021b
	P110	DRP1/Fis1 inhibitor		Guo X. et al., 2013
	HV3	Blocks the Htt/VCP interaction	Reduces excessive mitophagy	Guo et al., 2016
	DA1	Blocks the ATAD3A/DRP1 interaction	Rescues mitochondrial morphology	Zhao Y. et al., 2019
Amyotrophic lateral sclerosis	P110	DRP1/Fis1 inhibitor	Rescues mitochondrial morphology	Joshi et al., 2018b
	Olesoxime	Mitochondrial permeability transition pore inhibitor	Inhibits mitophagy	Bordet et al., 2007; Martin, 2010
	Nortriptyline	Mitochondrial permeability transition pore inhibitor	Inhibits mitochondrial permeability transition	Wang et al., 2007
	Resveratrol	Mitophagy activator	Enhances the clearance of damaged mitochondria	Markert et al., 2010; Mancuso et al., 2014; Laudati et al., 2019

## Mitochondrial Quality Control

### Homeostasis of the Mitochondrial Proteome

Mitochondria contain 1100–1300 proteins encoded by either the mitochondrial genome or nuclear genes (Calvo and Mootha, 2010). Under the regulation of particular synchronization programs, mitochondrial- and nuclear-encoded proteins coordinate exquisitely to sustain mitochondrial functions (Couvillion et al., 2016; Soto et al., 2021). Disrupting mitochondrial import and translation of mitochondrial- and nuclear-encoded proteins could affect the precise stoichiometry of the OXPHOS complex subunits and lead to proteotoxicity (Gomes et al., 2013; Houtkooper et al., 2013; Tang et al., 2020). The accumulation of mitochondrial proteome damage (e.g., misfolded protein and protein carbonylation) directly impacts mitochondrial integrity and aging. Therefore, quality control mechanisms are required to maintain mitochondrial protein homeostasis.

Mitochondrial proteostasis is surveilled and regulated by chaperone proteins and proteases (Moehle et al., 2019). Mitochondrial heat shock protein 70 (mtHSP70), mtHSP90, and the large chaperonin complex HSP60/10 correct protein folding (Mayer and Bukau, 2005; Saibil, 2013). In the mitochondrial matrix, Lon peptidase 1 (LONP1) (mammalian) and endopeptidase Clp (ClpP) recognize and degrade misfolded and damaged proteins (Haynes et al., 2007; Shin et al., 2021). The IMM contains both m-AAA (AFG3-like matrix AAA peptidase subunit [AFG3L2] and hereditary spastic paraplegia type 7 [SPG7]) and i-AAA (YME1L1) protease complexes face the matrix and degrade the proteins in the intermembrane space (IMS) (Stiburek et al., 2012; Shanmughapriya et al., 2015; Pareek and Pallanck, 2020).

**FIGURE 1:**
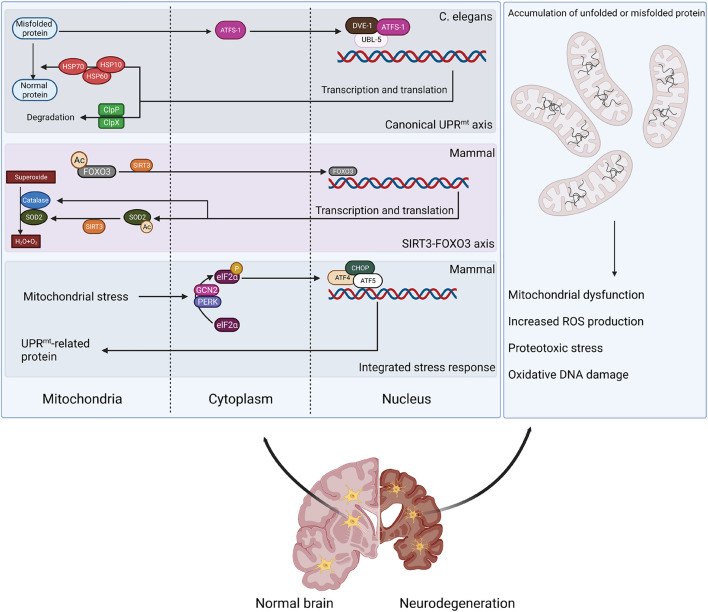
Retrograde signaling between mitochondria and nucleus to alleviate mitochondrial proteo-stress. Mitochondrial stress can stimulate defensive responses through different pathways. Canonical UPR^mt^ activation is mediated by ATFS-1 and transcription co-factors DVE-1/UBL-5 to stimulate expression of mitochondrial chaperones (HSP10/60/70) and proteases (ClpP). Proteo-stress-induced production of mitochondrial ROS activates SIRT3 to deacetylate FOXO3, stimulating antioxidant responses. Mitochondrial stress also triggers ISR by stimulating eIF2α phosphorylation by GCN2 or PERK and activating ATF4/ATF5/CHOP-induced transcription of UPR^mt^-related genes. However, mitochondrial stress response is defective in neurodegenerative disease, leading to a disturbance in protein homeostasis and mitochondrial dysfunction.

Unfolded protein response is one of the major mechanisms for maintaining mitochondrial proteostasis; its pathogenic relevance has recently been identified in NDs (Figure 1). UPR^mt^ is the retrograde signaling between the mitochondria and nucleus that induces mitochondrial proteases and chaperones, which alleviate an overload of mitochondrial proteins (Zhao et al., 2002; Aldridge et al., 2007; Nargund et al., 2012). Conditions that increase mitochondrial proteotoxicity (e.g., mitochondrial DNA [mtDNA] depletion or OXPHOS perturbation) can provoke UPR^mt^ (Lin et al., 2016; Naresh and Haynes, 2019). Though with some discrepancies, UPR^mt^ has been demonstrated in both Caenorhabditis elegans (*C. elegans*) and mammalian systems. In *C. elegans*, UPR^mt^ activation is mediated by activating transcription factor associated with stress-1 (ATFS-1) (Nargund et al., 2012). ATFS-1 has an amino-terminal mitochondrial-targeting sequence (MTS), which enables cells to evaluate mitochondrial protein import efficiency (Nargund et al., 2012). In cells with a healthy mitochondrial network, ATFS-1 is imported into mitochondria and degraded by the matrix-localized protease LON (Nargund et al., 2012). When mitochondria are impaired, the N-terminal nuclear localization signal (NLS) prevails and directs ATFS-1 to the nucleus to regulat transcription (Nargund et al., 2012). Importantly, MTS removal or inactivation results in the constitutive nuclear accumulation of ATFS-1 and UPR^mt^ activation (Nargund et al., 2012). Other transcription co-factors also mediate UPR^mt^ in *C. elegans*, including ubiquitin-like protein 5 (UBL5)/DVE-1 (Benedetti et al., 2006; Haynes et al., 2007). UPR^mt^ activation stimulates the expression of mitochondrial protease CLPP-1 and chaperones (e.g., mtHSP70) that are transported into the mitochondria to relieve proteo-stress (Haynes et al., 2007). Interestingly, chromatin remodeling is required for UPR^mt^ regulation in *C. elegans* (Merkwirth et al., 2016; Tian et al., 2016).

The regulation of UPR^mt^ is likely more complicated in mammalian cells. Recent studies suggest that three bZIP transcription factors (C/EBP homologous protein [CHOP] and activating transcription factors 4 and 5 [ATF4 and ATF5]) trigger UPR^mt^ activation, which requires the integrated stress response (ISR)-associated phosphorylation of translation initiation factor 2A (eIF2α) by general control non-derepressible 2 (GCN2) and protein kinase R (PKR)-like endoplasmic reticulum kinase (PERK) (Aldridge et al., 2007; Fiorese et al., 2016; Fiorese and Haynes, 2017; Kaspar et al., 2021). A recent study indicated that ATF5 is the mammalian ortholog of ATFS-1 because its activity appears to be regulated by mitochondrial import efficiency (Fiorese et al., 2016). During mitochondrial stress, ATF5 is required to induce multiple mitochondrial proteases and chaperones, including HSP60, mtHSP70, and LONP1 (Fiorese et al., 2016). UPR^mt^ activation can also be regulated by c-Jun N-terminal kinase (JNK) and the c-JUN pathway in mammals (Horibe and Hoogenraad, 2007; Nargund et al., 2012; Fiorese et al., 2016). The perturbation of protein homeostasis in the IMS can also stimulate the protective response mediated by mitochondrial sirtuin 3 (SIRT3) (Tseng et al., 2013; Qureshi et al., 2017). SIRT3 deacetylates and activates mitochondrial transcription factor forkhead box O3 (FOXO3), which then translocates to the nucleus to induce the expression of antioxidant enzymes (e.g., superoxide dismutase 2 [SOD2] and catalase) and biogenesis regulator peroxisome proliferator-activated receptor-γ coactivator-1α (PGC-1α) (Kenny et al., 2017; Weng et al., 2020). As a result, UPR^mt^ activation stimulates mitochondrial biogenesis and promotes pathogen resistance and lifespan (Qureshi et al., 2017).

Mitochondrial proteostasis and biogenesis can also be indirectly regulated by the master transcription co-factor PGC-1α. PGC-1α modulates the nuclear respiratory factors (NRF1 and NRF2) that regulate the expression of the ETC subunits encoded by the nuclear genome and bind to the promoter of genes involved in mtDNA transcription (Scarpulla, 2008), including mitochondrial transcription factor A (TFAM) (Virbasius and Scarpulla, 1994). In addition, NRF2 can regulate the expression of other mitochondrial enzymes, such as translocase of the outer membrane (TOM20) that mediates mitochondrial protein import (Blesa and Hernandez-Yago, 2006). PGC-1α is considered a neuroprotective target because a pathogenic role for PGC-1α dysregulation has been ubiquitously found in NDs (i.e., AD, PD, HD, and ALS) (Qin et al., 2009; Zheng et al., 2010; Johri et al., 2012; Thau et al., 2012). In parallel with the UPR^mt^, other quality control strategies (e.g., mitochondrial precursor over-accumulation stress [mPOS], the unfolded protein response activated by the mistargeting of proteins [UPR^*am*]^, mitochondrial ISR, and mitochondria-associated degradation [MAD]) have been implicated in regulating mitochondrial proteostasis and mitochondria-cytosol homeostasis (Silva et al., 2009; Wang and Chen, 2015; Wrobel et al., 2015; Liao et al., 2020). The pathological implications of these additional mechanisms in NDs are still under investigation. Thus, we mainly focus on UPR^mt^ in NDs in this review.

### Mitochondrial Fission and Fusion

Mitochondria are dynamic organelles that undergo continuous fission and fusion and are distributed in a tubular network in the cytoplasm (Giacomello et al., 2020). Mitochondrial fission segregates damaged parts from healthy mitochondria, whereas mitochondrial fusion allows the union of two mitochondria to enable genetic complementation, resulting in healthy and functional mitochondria (Ni et al., 2015; Giacomello et al., 2020). Thus, the balance between fission and fusion is critical for regulating mitochondrial size, number, and transport during cell proliferation and differentiation. It also ensures mitochondrial integrity coupled with appropriate bioenergetics to adapt to cellular stress.

**FIGURE 2:**
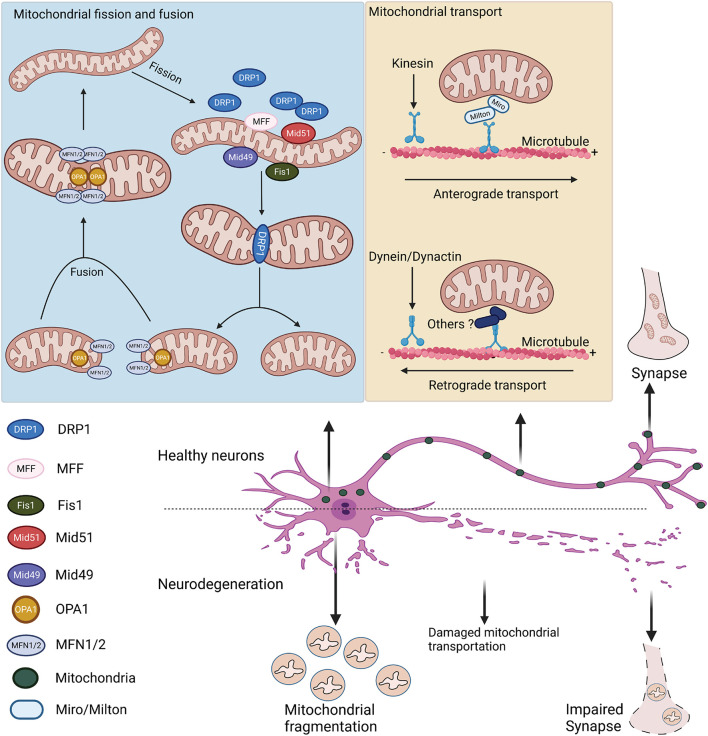
Mitochondrial fission, fusion, and transport in healthy and degenerative neurons. Mitochondrial fusion is mainly mediated by MFN1/2 and OPA1 on the outer and inner mitochondrial membranes, respectively. Mitochondrial fission is regulated by cytosolic DRP1 and its adaptor proteins (e.g., MFF, Fis1, and Mid49/51) on the OMM. After translocating onto mitochondria, DRP1 assembles to constrict the mitochondrial membrane. Mitochondrial axonal transport is mediated by the motor-adaptor protein complex. Anterograde transport is regulated by kinesin motor and Miro/Milton adaptors, whereas retrograde transport is regulated by the dynein/dynactin protein complex. In neurodegenerative diseases, dysregulated mitochondrial dynamics leads to mitochondrial fragmentation and impaired mitochondrial transport in neurons.

Mitochondrial fission and fusion are regulated by the evolutionally conserved GTPase-dynamin superfamily distributed in the cytosol, mitochondrial outer membrane (OMM), and IMM (Figure 2). Mitochondrial fusion requires the cooperation of the mitofusins (MFN1 and MFN2) and optical atrophy 1 (OPA1) (Cipolat et al., 2004; Song et al., 2009; Gao and Hu, 2021). MFN1 and MFN2 are located on the OMM and coordinate the fusion of the outer membrane (Schrepfer and Scorrano, 2016). OPA1 mediates the inner membrane fusion (Mishra et al., 2014). As the central mediator of mitochondrial fusion, OPA1 exists as long and short forms mediated by alternative splicing or processed by the IMM metalloendopeptidases OMA1 and YME1L1 (Anand et al., 2014; Baker et al., 2014; Gilkerson et al., 2021). In addition, OPA1 plays an independent role in maintaining the cristae structure (Patten et al., 2014). Mitochondrial fission relies on GTPase dynamin-related protein 1 (DRP1). Upon stimulation, this cytosolic protein is recruited onto mitochondrial surface, where it self-assembles into a spiral structure to constrict mitochondrial tubules, mediating division (Taguchi et al., 2007; Kalia et al., 2018). The recruitment of DRP1 onto the mitochondrial surface requires adaptor proteins residing on the OMM. While mitochondrial fission factor (MFF) mainly governs the mid-zone fission for biogenesis of new mitochondria, mitochondrial fission 1 (Fis1) regulates the peripheral fission for lysosomal degradation of damaged mitochondria (Kleele et al., 2021). The adaptor proteins (e.g., MFF and mitochondrial elongation factors MiD49/51) facilitate the recruitment of DRP1 to the OMM. Depletion of any of these adaptors results in elongated mitochondrial morphology, which mimics the effects of DRP1 depletion (Loson et al., 2013). There are indications that MFF and MiD49/51 interact to form a complex that mediates DRP1-dependent fission (Palmer et al., 2011). Mitochondrial fission sites appear to be determined by the physical interaction between the endoplasmic reticulum (ER) and mitochondria at the contact site (Friedman et al., 2011; Korobova et al., 2013). Live-cell imaging has demonstrated that the ER tubules cross and wrap around the mitochondria at the fission site, where MFF, MiD49/51, and DRP1 are often colocalized (Friedman et al., 2011). The site for mitochondrial fission is also influenced by mtDNA replication (Kraus and Ryan, 2017). Evidence has shown that mitochondrial nucleoids active in gene replication are highly associated with the constriction, leading to mitochondrial fission (Murley et al., 2013; Lewis et al., 2016). In cells, the site of mitochondrial fission is close to the mitochondrial nucleoids (Lewis et al., 2016).

In addition to fission and fusion, intracellular mitochondrial dynamics usually involve the transport and re-distribution of mitochondrial units, which is critical in neuronal cells that are polarized with long axons and dendrites (Yu and Pekkurnaz, 2018). Neuronal mitochondria are commonly found at the synaptic terminals, where they provide sufficient ATP and regulate Ca^2+^ for neurotransmission (Sheng and Cai, 2012). The bidirectional transport of mitochondria is coordinated by microtubule-based machinery and the result of mitochondrial coupling to motor-adaptor-receptor protein complexes (Saxton and Hollenbeck, 2012; Figure 2). Typically, anterograde transport of mitochondria toward the (+) end of the microtubules is mainly facilitated by motor proteins of the kinesin-1 families (Wang and Schwarz, 2009; Mandal and Drerup, 2019). The attachment of motor proteins to mitochondria is mediated by the adaptor proteins Milton and Miro, a mitochondrial Rho-like GTPase (Tang, 2016; Eberhardt et al., 2020). Retrograde mitochondrial transport is regulated by dynein motor protein and dynactin adaptor protein TRAK (kinesin binding protein) (Schnapp and Reese, 1989; Loss and Stephenson, 2017). The detailed mechanism of how the dynein motor binds to mitochondria is still largely unknown. After transport, mitochondria are stabilized on the axons by anchor protein syntaphilin, whose downregulation results in an increased percentage of mobile mitochondria along the axon (Lin et al., 2017; Cardanho-Ramos et al., 2020). Notably, Miro participates in mitochondrial axonal transport and facilitates mitochondrial fission, fusion, and Ca^2+^ homeostasis (Eberhardt et al., 2020). Recent studies using super-resolution microscopy identified Miro localization at the mitochondria- and ER-associated membrane (MAM), associated with the mitochondrial contact site and cristae organizing system (MICOS) (Modi et al., 2019). Though the detailed mechanisms need further investigation, these data suggest that Miro regulates mitochondrial fission and couples MICOS to the TRAK motor protein adaptors to ensure the transport of mitochondria. Intriguingly, Miro interacts with MFN2 to regulate mitochondrial fusion and axonal transport (Misko et al., 2010). The close relationship between mitochondrial fission and fusion and mitochondrial transport is noteworthy. The mitochondrial fission-related protein DRP1 is also implicated in mitochondrial transport. DRP1 inhibition sufficiently disrupts mitochondrial transport *in vitro* and *in vivo* (Mandal and Drerup, 2019). DRP1 is also important for the distribution of mitochondria in nerve terminals of dopaminergic neurons (Berthet et al., 2014). In addition, one recent study showed that DRP1 interacts with the dynein-dynactin complex to modulate retrograde mitochondrial transport. Moreover, MFN2 can directly regulate mitochondrial axonal transport in neurons, as MFN2 downregulation causes a decreased rate of mitochondrial transport and increased pausing time (Misko et al., 2010). Previous studies suggested that MFN2 is the direct receptor of the TRAK1/2 adaptor protein mediating mitochondrial transport (Lee C.A. et al., 2018). Moreover, inhibition of MFN2 in neurons and *in vivo* significantly reduces both anterograde and retrograde mitochondrial transport (Misko et al., 2010; Mandal and Drerup, 2019).

### Mitophagy

**FIGURE 3:**
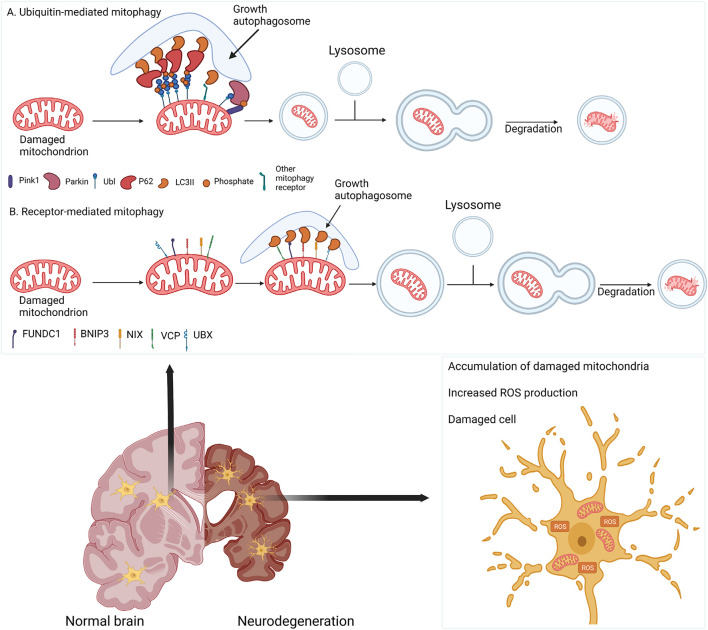
Damaged mitochondria are cleared by mitophagy. **(A)** Depolarized mitochondria retain PINK1 and Parkin to ubiquitinate OMM proteins, recruiting adaptor proteins (e.g., p62) to form autophagosomes that fuse with lysosomes for degradation. **(B)** In parallel, mitophagy can be mediated by receptor proteins (e.g., FUNDC1, BNIP3 and VCP) to recruit an autophagy adaptor. In neurodegenerative diseases, impaired mitophagy causes the accumulation of damaged mitochondria, increased ROS production, and neuronal death.

Mitophagy is the selective targeting and degradation of impaired mitochondria *via* lysosomes. It promotes the turnover of healthy mitochondria, regulates the number of mitochondria to meet metabolic demands, and eliminates dysfunctional mitochondria to prevent ROS production and the release of damage-associated patterns from mitochondria, which would further induce cellular stress and degeneration (Palikaras et al., 2018; Wang Y. et al., 2019). Mitophagy is regulated by several pathways in mammalian cells (Figure 3). PINK1-dependent Parkin activation is the best-characterized mitophagy pathway. In the healthy state, PINK1 is recruited and imported into mitochondria through the TOM-TIM (translocase of the inner membrane) complex, where it is cleaved by matrix processing peptidase (MPP) and presenilins-associated rhomboid-like protein (PARL) (Greene et al., 2012; Lazarou et al., 2012; Thomas et al., 2014; Kazlauskaite and Muqit, 2015; Pickrell and Youle, 2015). However, loss of mitochondrial membrane potential in dysfunctional mitochondria prevents the import of PINK1 and stabilizes it on the OMM, resulting in the phosphorylation of ubiquitin molecules (Greene et al., 2012; Pickles et al., 2018). Phosphorylated ubiquitin then recruits cytosolic Parkin to the mitochondria and activates its ubiquitin ligase activity (Kane et al., 2014; Pickles et al., 2018). Activated Parkin ubiquitinates OMM proteins, including voltage-dependent anion channel (VDAC) and mitofusins (Gegg et al., 2013; Yamano et al., 2016; McLelland et al., 2018; Ham et al., 2020), and recruits autophagy receptors (e.g., optineurin [OPTN], Tax1-binding protein [TAX1BP1], and sequestosome 1 [SQSTM1, or p62]), leading to the formation of microtubule-associated protein 1A/1B-light chain 3 (LC3)-positive phagophores (Geisler et al., 2010; Richter et al., 2016; Whang et al., 2017). Phagophores sequester damaged mitochondria and deliver them to the lysosomes for degradation. Recent findings suggested an emerging role for Miro in PINK1/Parkin-mediated mitophagy. It has been proposed that Miro is phosphorylated by leucine-rich repeat kinase-2 (LRRK2) in the presence of mitochondrial dysfunction, followed by the phosphorylation of both Miro and Parkin by PINK1 (Wang et al., 2011; Hsieh et al., 2016). Once activated, Parkin can ubiquitinate Miro, targeting it for proteasomal degradation and subsequent mitochondrial clearance by mitophagy (Panchal and Tiwari, 2021). In addition to PINK1 and Parkin, other receptor proteins can also mediate mitophagy. Notably, the AAA + ATPase valosin-containing protein (VCP)/p97 has emerged as a critical mitophagy receptor required for OMM-associated degradation and is involved in Parkin-dependent mitophagy (Sun and Qiu, 2020). Following mitochondrial depolarization, UBX domain-containing co-factor UBXN1/UBXD1 translocates alongside VCP to mitochondria in a Parkin-dependent manner and recruits LC3 to form phagophores (Bento et al., 2018; Mengus et al., 2021). VCP overexpression results in mitochondrial fragmentation and cell death (Fang et al., 2015). Furthermore, VCP mutations are associated with mitochondrial depolarization, oxidative stress in ALS and frontotemporal dementia (FTD) (Kakizuka, 2008; Meyer et al., 2012). FUN14 domain-containing-1 (FUNDC1) is an OMM protein that functions as a mitophagy receptor upon mitochondrial uncoupling and hypoxia (Zhang, 2021). BCL2-interacting protein-3 (BNIP3) is also involved in hypoxia-induced mitophagy (Ney, 2015).

## Alzheimer’s Disease

Alzheimer’s disease is the most common neurodegenerative disorder characterized by progressive memory loss and cognitive impairment, affecting 30 million people worldwide (Haque and Levey, 2019). The most prevalent pathological hallmarks of this disease include the accumulation of Aβ plaques and neurofibrillary tangles composed of misfolded microtubule-associated protein Tau. There is currently no cure for AD. Recent studies suggest that AD is not a linear downstream consequence of Aβ deposition but rather a multifactorial disease. In addition to Aβ toxicity, mitochondrial dysfunction has been suggested as a hallmark of AD because patients exhibit early metabolic changes (Moreira et al., 2010; Ortiz and Swerdlow, 2019). Furthermore, abnormal mitochondrial structure, accumulation of damaged mitochondria, and excessive ROS production are well-documented in various AD models (Ortiz and Swerdlow, 2019). Thus, impaired mitochondrial integrity might play an important role in AD pathogenesis. Understanding the mechanisms of mitochondrial dysregulation and impaired MQC may provide molecular targets for treating AD.

### Mitochondrial Quality Control Impairment in Alzheimer’s Disease

Previous studies demonstrated an imbalance between mitochondrial fusion and fission in AD, contributing to disease pathogenesis. Multiple independent studies have found that in AD patient brains and mutant amyloid precursor protein (APP)-expressing AD animal models, the expression of the mitochondrial fission-related protein Fis1 and the GTPase activity of DRP1 are increased (Cho et al., 2009; Wang X.L. et al., 2009; Joshi et al., 2018a). Moreover, the protein levels of fusion-related proteins (e.g., MFN1, OPA1, and MFN2) are decreased (Wang X. et al., 2009). Increased expression of mitochondrial fission-related proteins is correlated with Tau protein accumulation in neurons derived from AD patient-induced pluripotent stem cells (iPSCs) (Wang X. et al., 2008; Lee J. et al., 2018). The mRNA expression levels for genes expressing mitochondrial fission-related proteins (e.g., Fis1) are also increased in the peripheral blood of AD patients brain (Pakpian et al., 2020). Moreover, treatment of hippocampal neurons with Aβ-oligomer can reduce mitochondrial fusion (Wang X.L. et al., 2009). Furthermore, overexpression of either WT APP or mutant APPswe (APP Swedish mutant) can alter mitochondrial morphology and induce mitochondrial fragmentation in AD cell culture models (Wang X.L. et al., 2008). In addition to neurons, astrocytes carrying APOE3/4 variants, which is strong risk factor of AD, have altered mitochondrial DRP1 (Schmukler et al., 2020).

Impaired axonal transport is a featured axon pathology in AD. Both Aβ and Tau can disrupt axonal mitochondrial transport (Zheng et al., 2019). For example, Aβ treatment reduces mitochondrial mobility in hippocampal neuronal cultures (Zhao C. et al., 2019), and Tau overexpression in Neuro-2a cells disrupts axonal mitochondrial trafficking (Ebneth et al., 1998). Intriguingly, anterograde mitochondrial transport is more vulnerable in AD, possibly because Tau inhibits kinesin-1 activity but has little effect on dynein (Ebneth et al., 1998). Moreover, the mitochondrial anchor protein syntaphilin is degraded in AD-related human APP-expressing neurons, triggering retrograde transport of mitochondria (Lin et al., 2017). These results suggest that mitochondrial dynamics and mitochondrial transport are impaired in AD, which may lead to synaptic dysfunction and neurodegeneration. Furthermore, the accumulated mitochondria in the soma of neurons cannot be cleared by autophagy due to the defective transport and exacerbate neuronal loss in AD. These findings indicate a contribution of dysregulated mitochondrial dynamics and axonal transport to AD pathogenesis.

Mitophagy impairment is associated with ROS production, synaptic damage, Aβ accumulation, and neuronal loss in AD (Tran and Reddy, 2020). Mitophagy defects have been widely observed in various animal and cellular mutant APP-associated AD models. In a clinical study, the expression of Parkin and autophagy-related 5 (ATG5) was slightly but significantly decreased in the sera and brains from 160 AD patients compared to 40 control subjects (Castellazzi et al., 2019). In Tau and APP-expressing neuroblastoma cells, overexpression of P301L mutant Tau or mutant APPswe impairs the translocation of mitochondrial Parkin, PINK1, LC3-II/I, and other mitophagy-related proteins (Ye et al., 2015; Cummins et al., 2019). Mutation in presenilin-1 (PS1) causes familial AD. Mitophagy is impaired in the presenilin-1 (PS1) missense mutation A246E AD model due to increased lysosomal pH (Coffey et al., 2014). In AD patient iPSC-derived neuronal cells, the expression levels of LC3 and transcription factor EB (TFEB) are decreased (Martin-Maestro et al., 2019). Thus, a reduction in mitophagy-related proteins or changes in the cellular environment can result in impaired mitophagy.

Disruptions in mitochondrial proteostasis have been repeatedly observed in AD. Aβ can be translocated into mitochondria, interrupting mitochondrial protein import and impairing preprotein maturation. These disturbances lead to mitochondrial dysfunction (Mossmann et al., 2014; Cenini et al., 2016). Devi et al. (2006) demonstrated that both full-length and C-terminal truncated APP could accumulate in the mitochondrial protein import channels (TOM40 and TIM23), causing mitochondrial protein import deficits and dysfunction exclusively in AD patient brains. In a yeast model, mitochondrial Aβ peptide inhibited the degradation of presequence peptides by presequence protease (PreP) homolog Cym1, leading to the accumulation of mitochondrial preprotein and processing intermediates and an imbalanced organellar proteome (Mossmann et al., 2014). The activation of UPR^mt^ was recently reported in AD patients and other models, which may disrupt proteostasis through Aβ or APP. Accumulation of mitochondrial unfolded protein has been observed in AD patient brains and is a potential diagnostic biomarker for this disease (Beck et al., 2016). The mRNA levels of UPR^mt^-related components (e.g., *DNAJA3, HSPD1, HSPE1, CLPP*, and *YME1L1*) are all significantly increased in AD subjects (Beck et al., 2016). In addition, HSP60 protein levels are increased in AD patients, possibly resulting from the constitutive activation of the mitochondrial stress response (Walls et al., 2012). One recent study demonstrated that UPR^mt^ responses are associated with Aβ toxicity in AD patients, mouse and *C. elegans* models of AD (Sorrentino et al., 2017). Notably, pharmaceutically or genetically improved mitochondrial proteostasis reduced amyloid aggregation in *C. elegans* AD model, and a transgenic AD mouse model, indicating a protective role for UPR^mt^ in AD (Sorrentino et al., 2017). In Aβ peptide (Aβ_25–35_)-treated neuroblastoma cells and APPswe/PS1dE9 double transgenic mice, the HSP60, LONP1 and ClpP expression levels are elevated compared to the controls (Shen et al., 2019). The connection between UPR^mt^ and Alzheimer’s disease was further demonstrated in PreP knock-out brain organoids, where a mitochondrial PreP deficiency induced UPR^mt^, increased Aβ accumulation, and triggered AD-like phenotypes (Perez et al., 2020). Further studies are needed to understand how APP or Aβ induces UPR^mt^ activation and disrupts mitochondrial proteostasis in AD.

### Targeting Mitochondrial Quality Control Prevents Alzheimer’s Disease-Associated Pathology

Mitochondrial quality control maintains mitochondrial function. In AD patients and model systems, expression levels of MQC components involved in mitochondrial dynamics, mitophagy, and UPR^mt^ are significantly altered. Restoration or overexpression of these components may be possible therapeutic approaches for AD. Multiple evidences suggest the abnormal mitochondrial fission in AD patient and animal and cellular models of AD, inducing mitochondrial fragmentation (Calkins et al., 2011; Zhang L. et al., 2016). Thus, the inhibition of fission-related proteins or overexpression of fusion-related proteins may represent possible treatments (Xie et al., 2014). Several compounds can reduce the expression levels of mitochondrial fission-related proteins or induce fusion-related proteins. DRP1 inhibitor, mitochondrial division inhibitor-1 (Mdivi1), and the mitochondria-targeted antioxidant Szeto-Schiller tetrapeptide 31 (SS31) reduced the levels of hydrogen peroxide and GTPase DRP1 activity in mutant APPswe-overexpressing Neuro-2a cells (Reddy et al., 2017b, 2018). In senescence-accelerated mouse-prone 8 (SAMP8) mice that have an accelerated aging phenotype, SS31 can correct learning disabilities by decreasing the levels of the mitochondrial fission proteins DRP1 and Fis1 (Jia et al., 2016). Mdivi1 can attenuate mitochondrial fission, reverse the inhibition of mitochondrial complex I, and modulate reactive oxygen species levels by impairing DRP1 GTPase activity in Aβ-treated BV-2 microglia cells (Park et al., 2013). Treatment with the mitochondria-targeted hydrogen sulfide donor AP39 attenuates mitochondrial impairment by increasing OPA1 and MFN1 expression levels and decreasing Fis1 protein levels in APP/PS1 neurons and transgenic mice (Zhao et al., 2016). In addition, administration of citalopram, a selective serotonin reuptake inhibitor, can alleviate memory loss, cognitive decline, defective biogenesis, impaired dendritic spines and defective synaptic MQC in APP/PS1 double transgenic mice (Zhang et al., 2018). This compound also activates mitochondrial fusion and reduces mitochondrial fission (Zhang et al., 2017). Glycogen synthase kinase 3 (GSK3)-dependent phosphorylation of DRP1 at Ser40 and Ser44 can increase DRP1 GTPase activity, which induces mitochondrial fragmentation (Yan et al., 2015). The synthetic polypeptide TAT-DRP1-SpS can block this phosphorylation and reduce mitochondrial fragmentation in the APP/PS1 AD mouse model (Yan et al., 2015). DDQ and SS31 can enhance mitochondrial fusion by activating MFN1 and MFN2 and repress Bax activation, cytochrome c release, and the mitochondrial permeability transition pore in APPswe-expressing cells (Kuruva et al., 2017). DRP1 siRNA can also prevent mitochondrial fission, loss of mitochondrial membrane potential, and cell death (Grohm et al., 2012). Zhang et al. (2020) showed that conditional heterozygous depletion of DRP1 in oligodendrocytes could rescue mitochondrial morphology, abolish NLR family pyrin domain containing 3 (NLRP3)-mediated inflammatory injury, and restore axonal myelination in the 5xFAD AD mouse model. Rescue of mitochondrial transport may also have a beneficial effect in AD. Indeed, blockage of the mitochondrial permeability transition pore by genetic depletion of cyclophilin D or treatment with SS31 attenuates impaired mitochondrial trafficking, potentially due to reduced Ca^2+^ and ROS (Guo L. et al., 2013; Jia et al., 2016).

Enhanced mitophagy-related protein levels in mouse and cellular AD models can restore mitophagy and reduce neuronal stress caused by the accumulation of damaged mitochondria. For example, treatment of APP/PS1 mice with the phosphodiesterase (PDE)-7 inhibitor S14 has a neuroprotective effect by modulating Aβ-induced mitochondrial dysfunction through restored LC3-II expression levels (Bartolome et al., 2018). In addition, citalopram activates PINK1, ATG5, ATG7, p62, and LC3B-I/II, restoring mitophagy and abrogating synaptic toxicities in APP-Tg2576 transgenic mice (Reddy et al., 2021). Parkin overexpression in APP/PS1 mice and Aβ-treated cells can ameliorate mitochondrial dysfunction, restore PINK levels, increase ATP production, and decrease ubiquitinated Aβ levels (Hong et al., 2014; Martin-Maestro et al., 2016; Wang H.M. et al., 2020). Moreover, nicotinamide riboside (NR) increases mitophagy-related proteins (e.g., LC3) to enhance mitophagy and alleviate memory loss in APPswe/PS1dE9 AD mice (Aman et al., 2020). Furthermore, Fang et al. (2019) demonstrated that treatment with the mitophagy activator urolithin A restored mitophagy by increasing PINK1, Parkin, and Beclin-1 protein levels and attenuates the loss of cognitive ability and Aβ pathology in APP/PS1 mice. In the familial AD-related mutant PS1 iPSC-derived neural stem cells, bexarotene can restore mitophagy and rescue the damaged mitochondrial network morphology (Martin-Maestro et al., 2019). Treatment of *C. elegans* co-expressing Aβ and Tau with spermidine improved behavior, extend lifespan, and protect against memory loss *via* the PINK/Parkin pathway (Yang et al., 2020). Other enzymes (e.g., adenylate-activated protein kinase [AMPK] and SIRT) can also influence mitophagy activity. For instance, AMPK overexpression sufficiently reduces Tau phosphorylation, GSK3β activity, and brain impairment in streptozotocin (STZ) mice (Wang L. et al., 2020). In addition, SIRT activators (e.g., resveratrol) induce autophagy through the mTOR (Target of rapamycin)-ULK1 (Unc-51 Like Autophagy Activating Kinase 1) pathway (Park et al., 2016). Clinical trials have also shown that SIRT activators modulate Aβ levels and inflammatory markers in AD patients. Finally, compounds that can induce lysosomes and autophagosomes, such as trehalose, can also induce mitophagy and attenuate the accumulation of APP in AD (Tien et al., 2016).

Unfolded protein response maintains homeostasis and reduces the proteotoxicity caused by Aβ (Munoz-Carvajal and Sanhueza, 2020); its activation can remove unfolded proteins induced by APP mutants. Enhancement of UPR^mt^ can restore memory and reduce Aβ toxicity in the APP/PS1 double transgenic mice (Shen et al., 2020). Recent studies have shown that the sirtuin family is essential for UPR^mt^, and a reduction in SIRT3 expression correlates with mitochondrial dysfunction in AD (Lee J. et al., 2018). NAD^+^ can act as a UPR^mt^ inducer by activating SIRT3 (Papa and Germain, 2014). In *C. elegans*, an NAD^+^ booster could attenuate the proteotoxicity caused by Aβ (Mouchiroud et al., 2013). Induction of the expression of the chaperones mtHSP70 and mtHSP90 may inhibit Aβ aggregation and the formation of plaques in the AD brain (Roe et al., 2018; Evgen’ev et al., 2019).

## Parkinson’s Disease

Parkinson’s disease is the second predominant neurodegenerative disorder that affects more than 10 million people worldwide (Ball et al., 2019). PD is characterized by a selective loss of dopaminergic neurons in the substantia nigra, resulting in rest tremor, bradykinesia, rigidity, and the accumulation of intracellular Lewy bodies composed of α-Synuclein protein (α-Syn). Although the pathogenic mechanisms of PD remain elusive, numerous studies suggest a dominant role for mitochondrial dysfunction in various PD models. Notably, most of the PD-related mutations were identified in proteins that regulate mitochondrial functions. Thus, improving mitochondrial function by targeting MQC might efficiently ameliorate PD pathogenesis.

### Mitochondrial Quality Control Impairment in Parkinson’s Disease

Recent studies have demonstrated UPR^mt^ impairment in multiple PD models. Mutant α-Syn (e.g., A30P/A53T) is prone to aggregate and associated with early onset of PD (Li et al., 2001). In transgenic mice, human dopaminergic SH-SY5Y cells, and iPSC-derived dopaminergic neurons, the α-Syn A53T mutant can preferentially accumulate in mitochondria and interact with matrix ClpP, suppressing its peptidase activity. These events result in mitochondrial dysfunction and neuronal damage (Hu et al., 2019). Mutation in PINK1/PRKN is associated with familial PD. In nematode PD models, missense mutations in *pink-1*/*pdr-1* caused the accumulation of damaged mitochondria, which activated UPR^mt^ to mitigate the detrimental effects of these mutations (Cooper et al., 2017). Treatment of SH-SY5Y cells with mitochondrial toxin 1-methyl-4-phenylpyridinium (MPP +) (metabolite of 1-methyl-4-phenyl-1,2,3,6-tetrahydropyridine [MPTP]) that selectively impairs dopaminergic neuron by inhibiting ETC complex I impaired UPR^mt^ and induced OXPHOS stress, which could be reversed by overexpression of PGC-1α or ATF5 (Cai et al., 2020; Hu et al., 2021a).

Abnormal mitochondrial dynamics have been reported in PD. α-Syn has been shown to directly regulate mitochondrial morphology. α-Syn overexpression causes mitochondrial fragmentation in multiple PD models, including WT α-Syn-expressing *C. elegans*, A53T α-Syn-expressing SH-SY5Y cells, and primary neurons from A53T α-Syn-expressing rat (Kamp et al., 2010; Li et al., 2013; Bido et al., 2017). Recent studies have illustrated the molecular mechanisms underlying α-Syn-induced abnormalities in mitochondrial morphology. Oligomeric α-Syn binds to the lipids in the OMM and distresses membrane curvature, reducing mitochondrial fusion rate (Ryan et al., 2018; Gilmozzi et al., 2020). Moreover, WT α-Syn can localize to the MAM to positively modulate mitochondrial morphology. This function is impaired by pathogenic mutations (e.g., A53T) in α-Syn (Guardia-Laguarta et al., 2014). Overexpression of A53T α-Syn activates mitogen-activated protein kinase (p38 MAPK), which phosphorylates DRP1 at serine 616 (S616) and triggers mitochondrial fission *in vitro* (Gui et al., 2020). In addition, MFN1 and MFN2 protein levels are decreased in α-Syn-expressing primary cortical neurons, which correlates with a decrease in mitochondrial fusion and smaller mitochondria (Faustini et al., 2019). In a *Drosophila* model of α-Syn-associated PD, mutant α-Syn proteins misallocate to mitochondria where they interact with spectrin and alter actin stabilization, resulting in DRP1 recruitment, mitochondrial fragmentation, and neuronal death (Ordonez et al., 2018).

Increased α-Syn levels have also been linked to the arrest of both anterograde and retrograde mitochondrial transport (Prots et al., 2018; Valdinocci et al., 2019). The anterograde trafficking of mitochondria is disrupted in early-stage sporadic PD followed by altered retrograde transport in late-stage disease (Zanellati et al., 2015). Prots et al. (2018) showed that the E57K α-Syn variant tends to form oligomers that directly bind to and disrupt the interactions between kinesin and the microtubules in human neuronal cells. The α-Syn A53T mutant can also directly bind to Miro, causing aberrant mitochondrial axonal transport in human neurons and *Drosophila* (Shaltouki et al., 2018). Mutations in other genes that are risk factors for familial PD can also cause dysfunctional mitochondrial dynamics. Vacuolar protein sorting-associated protein 35 (VPS35) is a component of the retromer complex, which is involved in retrograde transport from endosomes to the trans-Golgi network (Tang et al., 2015). Loss of function mutations in VPS35 impair the proteostasis including the enlargement of endolysosomes and is linked with autosomal dominant PD (Vilarino-Guell et al., 2011). The VPS35 R524W and D620N mutants increase the clearance of inactive DRP1, leading to mitochondrial fragmentation in human neurons and mouse brain (Wang et al., 2016). LRRK2 is a kinase localized to the cytosol and associated with the OMM (Biskup et al., 2006). Mutation in LRRK2 has been linked to the late-onset of PD (Clark et al., 2006). The LRRK2 G2019S mutant can directly phosphorylate DRP1 at threonine 595 (Thr595) to cause excessive mitochondrial fission in iPSC-derived neurons (Su and Qi, 2013). Moreover, this mutant appears to alter the polymerization/depolymerization cycles of microtubules, disrupting mitochondrial trafficking in PD (Kett et al., 2012; Godena et al., 2014; Singh et al., 2019). LRRK2 and PINK1 *Drosophila* mutants exhibit disturbed mitochondrial calcium homeostasis with functional involvement of Miro (Lee K.S. et al., 2018). Mutant LRRK2 can also induce PINK1 and Parkin-dependent Miro degradation and mitophagy, which inhibits mitochondrial axonal transport (Bonello et al., 2019; Liu et al., 2019). MPP + induces Parkin S-nitrosylation, triggering DRP1 phosphorylation at S616 and excessive mitochondrial fission (Zhang Z. et al., 2016). Moreover, MPP + can inhibit kinesin-1-mediated anterograde mitochondrial transport (Kim-Han et al., 2011).

Mitophagy is impaired in numerous ways in PD. PINK1 and Parkin mutations resulting in loss of function lead to autosomal recessive forms of PD (Lucking et al., 2000; Morais et al., 2009; Vives-Bauza et al., 2010). In addition to mitophagy, PINK1 and Parkin are also important for maintaining mitochondrial morphology (Poole et al., 2008). In A53T α-Syn transgenic mice, α-Syn accumulation on mitochondria causes increased mitophagy and neuronal death (Choubey et al., 2011). In A53T and E46K α-Syn transgenic mice, accumulation of α-Syn on the mitochondria promotes externalization of cardiolipin from the IMM to OMM to recruit LC3 to the mitochondria, inducing mitophagy (Ryan et al., 2018). In the neurons derived from PD patient iPSCs, α-Syn interacts with Miro *via* its N-terminus, leading to excessive Miro accumulation on the mitochondrial surface and delayed mitophagy (Shaltouki et al., 2018). These studies indicate the role of abnormal mitophagy in α-Syn-mediated toxicity. In addition to PINK1/Parkin, LRRK2 depletion or the LRRK2 G2019S mutant impair the autophagy/lysosomal pathway, leading to the accumulation of autophagosomes (Bonello et al., 2019; Obergasteiger et al., 2020; Wauters et al., 2020; Boecker et al., 2021). The levels of the autophagy markers p62 and LC3 are increased in induced pluripotent stem cell-derived dopaminergic neurons from PD patients with the LRRK2 G2019S mutation (Vermilyea et al., 2020). Several independent studies have shown PINK1/Parkin-dependent accumulation of Ras-related protein Rab-10 (RAB10), an LRRK2 substrate, on damaged mitochondria in PD patients with the LRRK2 G2019S mutation, suggesting that LRRK2 is involved in PINK1/Parkin-mediated mitophagy (Bonello et al., 2019; Wauters et al., 2020). Mutant DJ-1 causes a rare form of autosomal recessive PD. DJ-1 is regarded as a neuroprotective factor that translocates onto stressed mitochondria to regulate the clearance of ROS. DJ-1 loss-of-function increases the recruitment of Parkin to damaged mitochondria and mitophagy activity (Thomas et al., 2011).

### Targeting Mitochondrial Quality Control Prevents Parkinson’s Disease-Associated Pathology

Given the importance of mitochondrial dysfunction in PD pathogenesis, therapeutics targeting mitochondria have been studied for the prevention and treatment of PD. Although the pathological phenotypes from mutation-associated and neurotoxin-induced PD models are different, the outcomes of mitochondrial dysfunction, including impaired mitochondrial dynamics, mitophagy, and UPR^mt^, are the same. Therefore, understanding the mechanisms of MQC impairment in PD could provide potential targets for developing novel PD treatments.

Several lines of evidence demonstrate the beneficial effects of UPR^mt^ activation in both familial and idiopathic PD models. In SH-SY5Y cells, upregulation of UPR^mt^ activity by overexpression of PGC-1α or ATF5 significantly improved mitochondrial function and cell survival after MPP + treatment (Cai et al., 2020; Hu et al., 2021a). UPR^mt^ activation by ATFS-1 protected *C. elegans* against mutant PINK1/Parkin-induced mitochondrial fragmentation, oxidative stress, and cellular toxicity, promoting longevity and dopaminergic neuron survival (Cooper et al., 2017). Moreover, activation of UPR^mt^ by ginseng total protein (GTP) from herbal extracts rescued PD-related pathologies in mutant PINK1^*B*9^-expressing *Drosophila* and prolonged their lifespan (Liu M. et al., 2020). Furthermore, the α-Syn A53T mutant preferentially accumulates in mitochondria and directly binds to UPR^mt^-related ClpP, suppressing its peptidase activity (Hu et al., 2019). Conversely, ClpP overexpression sufficiently decreases α-Syn A53T mutant-associated pathology (Hu et al., 2019).

Pharmaceutical and genetic approaches to modulate mitochondrial dynamics efficiently improve mitochondrial integrity and neuronal survival in PD. Filichia et al. (2016) showed that subcutaneous administration of rationally designed small peptide P110, a selective inhibitor of the DRP1/Fis1 interaction, to MPTP-treated mice blocked DRP1 mitochondrial translocation and protected dopaminergic neurons. Similarly, the small-molecule DRP1 inhibitor Mdivi-1 attenuated mitochondrial fragmentation and α-Syn aggregation and prevented motor deficits in the α-Syn A53T mutant-expressing rat model (Bido et al., 2017). Moreover, overexpression of MFN2 or a DRP1 K38A dominant-negative variant rescued mitochondrial deficits and neuropathology in the α-Syn A53T mutant rat model (Choubey et al., 2011; Rappold et al., 2014). 6-Hydroxydopamine (6-OHDA) is a neurotoxin that can selectively trigger dopaminergic neuronal loss (Simola et al., 2007). MitoQ is a mitochondria-targeted antioxidant that consists of a lipophilic triphenylphosphonium (TPP) cation linked to a ubiquinone antioxidant moiety of the endogenous antioxidant coenzyme Q10. In 6-OHDA-treated cells and a PD mouse model, MitoQ activated PGC-1α, enhancing MFN2-mediated mitochondrial fusion and the survival of dopaminergic neurons (Xi et al., 2018). Furthermore, LRRK2 inhibition can correct mitochondrial transport and morphology to preserve neuronal function in PD animal models (Xiong et al., 2017; Singh et al., 2019).

Because the pathogenic role of mitophagy defects in PD has been elucidated, improving the efficiency of mitochondrial clearance by mitophagy may represent a disease-modifying strategy for PD. Indeed, studies focusing on the development of mitophagy modulators have demonstrated their therapeutic potential in NDs. For instance, PINK1 can be targeted for cellular elimination through the ubiquitin E3 ligase subunit, F-box protein 7 (FBXO7) (Liu Y. et al., 2020). Indeed, Liu Y. et al. (2020) demonstrated that compound BC1464, which specifically disrupts the FBXO7/PINK1 interaction, could rescue mitophagy and confer neuroprotection in several PD culture models (e.g., primary cortical neurons, neuroblastoma cells, and patient-derived cells). Treatment with a Miro1 reducer (compound 3) decreased mitochondrial-localized Miro levels, rescuing mitochondrial transport and PD-related phenotypes in iPSC-derived neurons and *Drosophila* models (Hsieh et al., 2019). Parkin sulfhydration is markedly decreased in PD patients; however, the catalytic activity of Parkin can be improved through this modification, suggesting the therapeutic potential of hydrogen sulfide donors (Vandiver et al., 2013). A recent high-throughput screening for compounds that upregulate the mitochondrial recruitment of Parkin identified a series of neuroprotective Rho-associated protein kinase (ROCK) inhibitors (Moskal et al., 2020). In dopaminergic neurons and SH-SY5Y cells, activation of PINK1 with the ATP neo-substrate kinetin/kinetin triphosphate (KTP) significantly improves the activity of WT PINK1 and the PINK1 G309D mutant and enhances Parkin phosphorylation and its mitochondrial recruitment (Hertz et al., 2013). Ubiquitin-specific protease 30 (USP30) is a mitochondrial deubiquitinase that opposes Parkin-mediated mitophagy by removing the poly-ubiquitin chain from damaged mitochondria (Bingol et al., 2014). Knock-down of USP30 enhances mitophagy and rescues paraquat-induced dopamine neuronal loss in *Drosophila* (Bingol et al., 2014). In addition, two distinct USP30 inhibitors (FT385 and USP30i) can significantly improve mitophagy, indicating potential therapeutic approaches for PD (Bingol et al., 2014; Rusilowicz-Jones et al., 2020). Treatment of MPTP-treated mice with the mTOR activator rapamycin triggers mitophagy, preventing neuronal loss (Moors et al., 2017). Furthermore, genetic upregulation of PINK1 or Parkin can significantly alleviate MPTP-induced neurodegeneration and motor deficits in PD mice (Li and Chen, 2019).

## Huntington’s Disease

Huntington’s disease is a devastating monogenic neurological disorder caused by the dominantly inherited CAG trinucleotide repeat expansion in the gene encoding the huntingtin (Htt) protein (Kerschbamer and Biagioli, 2016). Clinical symptoms of HD include hyperkinesia or chorea of the face, trunk, and legs, followed by cognitive and psychiatric disturbances (Roos, 2010). HD is characterized by the selective loss of GABAergic medium spiny neurons (MSN) and the presence of mutant huntingtin (mtHtt) aggregates in the striatum (Ehrlich, 2012). With polyglutamine expansion at the N-terminus, mtHtt protein gains a toxic function that disturbs multiple subcellular functions, leading to neuronal death (Schulte and Littleton, 2011). Mitochondrial dysfunction has been widely demonstrated to be correlated with the loss of MSN and HD pathogenesis. Thus, enhancing mitochondrial function is a potential therapeutic strategy for HD.

### Mitochondrial Quality Control Impairment in Huntington’s Disease

Mutant huntingtin impairs mitochondrial function through multiple pathways. HD patients exhibit well-documented metabolic defects (Nambron et al., 2016). mtHtt represses the activity of PGC-1α, a transcriptional coactivator involved in mitochondrial biogenesis, glucose metabolism, β-oxidation of fatty acids, and adaptive thermogenesis (Cui et al., 2006). As a result, the activities of OXPHOS complexes I, II, III, and IV are affected in HD patient brains (Cui et al., 2006). Alternations in mitochondrial dynamics are well characterized in HD. Song et al. (2011) demonstrated that mtHtt could bind to and activate DRP1, leading to DRP1 mitochondrial translocation and mitochondrial fragmentation in HD patient brains and mice models. In addition to directly interacting with mtHtt, DRP1 can be activated by multiple kinases, including MAPK/ERK2 and cyclin-dependent kinase (CDK5) (Jahani-Asl et al., 2015; Roe and Qi, 2018). Moreover, mtHtt-induced DRP1 translocation can trigger the dimerization of ATPase Family AAA Domain Containing 3A (ATAD3A) in multiple neuronal and mouse models of HD, resulting in mitochondrial fragmentation and mtDNA damage (Zhao Y. et al., 2019). Interestingly, mtHtt can also affect mitochondrial movement when expressed in primary rat cortical neurons (Chang et al., 2006). Furthermore, the mtHtt binding partner huntingtin-associated protein (HAP1) can interact with kinesin and dynein to regulate mitochondrial transport (Rong et al., 2006).

It has recently been discovered that mtHtt can disrupt mitochondrial proteostasis in HD. mtHtt localizes to the IMS in mtHtt-expressing cells and HD patient brains where it binds with high affinity to the TIM23 complex, causing defective import of nuclear-encoded proteins and disrupting mitochondrial proteostasis (Yablonska et al., 2019). Moreover, mtHtt inhibits UPR^mt^ in HD cells and HD R6/2 transgenic mice by impairing the mRNA stability of mitochondrial ATP Binding Cassette Subfamily B Member 10 (ABCB10), which suppresses UPR^mt^ signaling (Fu et al., 2019). Several recent studies have suggested that the removal of defective mitochondria is compromised in HD. Indeed, mtHtt affects autophagosome formation, leading to the accumulation of damaged mitochondria (Lee et al., 2012; Franco-Iborra et al., 2021). While its physiological function is still unknown, glyceraldehyde-3-phosphate dehydrogenase (GAPDH)-mediated mitophagy is a novel micro-mitophagy pathway to eliminate damaged mitochondria *via* lysosomes following ischemia or reoxygenation-induced injury, which is independent of its glycolytic activity (Yogalingam et al., 2013). Interestingly, mtHtt can interact with mitochondrial GAPDH, which stalls GAPDH-mediated mitophagy and causes the accumulation of damaged mitochondria in HD cells (Hwang et al., 2015). One recent study also suggested a pathogenic role for excessive mitophagy in HD. In HD patients and transgenic mouse models, the interaction of VCP with mtHtt causes its translocation and accumulation in mitochondria, triggering excessive mitophagy via the recruitment of LC3 to the mitochondria (Guo et al., 2016). However, another study indicated that the interaction between mtHtt and autophagy receptor p62 disrupts the loading of damaged mitochondria into autophagosome and their transport to lysosomes (Ehrnhoefer et al., 2018).

### Targeting Mitochondrial Quality Control Rescues Huntington’s Disease-Associated Pathology

Many lines of evidence have shown that improving mitochondrial function can efficiently rescue HD-related pathology, indicating the role of mitochondrial dysfunction in HD pathogenesis and the therapeutic potential of targeting MQC. SIRT3 activation can improve anterograde mitochondrial neurite transport and maintain the viability of primary striatal neurons from HD mice (Naia et al., 2021). Furthermore, the overexpression of the SIRT3 ortholog dSirt2 ameliorated neurodegeneration and extended lifespan of HD flies (Naia et al., 2021). Inhibition of DRP1 mitochondrial translocation can sufficiently rescue mitochondrial morphology, biogenesis, and neuronal viability in HD models. Recently, Hu et al. (2021b) identified CHIR99021 as a mitochondrial enhancer that can significantly improve mitochondrial function (e.g., mitochondrial membrane potential, respiration) by preventing DRP1 translocation *via* calpastatin stabilization in HD neurons, and rescue the neuropathology and motor dysfunctions in HD mouse models. Moreover, inhibition of DRP1 activity by small molecule inhibitor Mdivi-1 or overexpression of the dominant-negative DRP1 K38A mutant prevents mitochondrial fission and improves mitochondrial function (Song et al., 2011; Manczak and Reddy, 2015). In addition, Guo X. et al. (2013) demonstrated that long-term administration of peptide P110, which interferes with the interaction between DRP1 and Fis1, can rescue mitochondrial fragmentation, HD-related neuropathology, and motor deficits observed in HD mouse models. Treatment with peptide inhibitor DA1 that blocks Drp1/ATAD3A interaction can rescue mitochondrial fragmentation and mtDNA lesion, and HD-related pathologies in HD mouse models (Zhao Y. et al., 2019). Thus, antagonizing DRP1-mediated mitochondrial fission could represent an important therapeutic approach against HD.

Approaches mediating mitophagy can also protect against HD. Khalil et al. (2015) showed that PINK1 overexpression could ameliorate ATP levels and improve neuronal integrity and survival in an HD *Drosophila* model, counteracting mtHtt toxicity. Subcutaneous administration of the small peptide HV3 to HD mice abolished the mitochondrial translocation of VCP by blocking the interaction between mtHtt and VCP (Guo et al., 2016). This treatment also corrected excessive mitophagy and reduced cell death. In addition, GAPDH overexpression is sufficient to rescue defective mitophagy, enhance mitochondrial function, and promote cell survival (Hwang et al., 2015). Moreover, treatment with mitochondrial activators that induce PGC-1α expression promotes mitochondrial biogenesis and provides neuroprotection by activating autophagy and increasing the turnover of mtHtt aggregates (Tsunemi et al., 2012).

## Amyotrophic Lateral Sclerosis

Amyotrophic lateral sclerosis is the most common subtype of motor neuron disease, with a worldwide prevalence of 4–6 in 100,000 people (Bucheli et al., 2013). It is an incurable, fatal neurodegenerative disorder with an average survival of 2-3 years from diagnosis (Bryukhovetskiy et al., 2020). ALS is characterized by rapid, progressive degeneration of upper and lower motor neurons, resulting in muscle atrophy, gradual paralysis, and death (Ravits et al., 2007). ALS is a multi-factorial disease. Proteins altered in ALS, such as superoxide dismutase 1 (SOD1), TAR DNA binding protein (TDP43), fused in sarcoma (FUS), and C9orf72, have been implicated in a wide range of cellular pathways (Bruijn et al., 1997; Neumann et al., 2006; Vance et al., 2009; Cooper-Knock et al., 2012). Notably, all these proteins can cause mitochondrial dysfunction. Thus, mitochondrial dysfunction is a crucial factor involved in ALS pathogenesis and rescuing mitochondrial integrity might protect motor neuron function.

### Mitochondrial Quality Control Impairment in Amyotrophic Lateral Sclerosis

Though still under investigation, evidence has shown UPR^mt^ activation in various ALS models. The SOD1 G93A mutant localizes to the IMS and activates two UPR^mt^ axes in an ALS mouse model (Riar et al., 2017). TDP-43 dysregulation suppresses ETC complex I and activates UPR^mt^ in cellular and mouse ALS models (Wang P. et al., 2019). Another study showed that TDP-43 is a potential substrate for UPR^mt^ protease LONP1, and downregulation of LONP1 increases TDP-43 expression, resulting in mitochondrial dysfunction and neurodegeneration (Wang P. et al., 2019). More recently, mutations have been reported in the coiled-coil-helix-coiled-coil-helix domain containing 10 (CHCHD10) gene in ALS patients (Veldink and Consor, 2018; Harjuhaahto et al., 2020). CHCHD10 encodes a mitochondrial protein, which may maintain the MICOS (Genin et al., 2016). Mutant CHCHD10 is associated with mitochondrial dysfunction and the early death of motor neurons (Ryan et al., 2021). In mutant CHCHD10 ALS mice, aggregation of mutant CHCHD10 induces proteotoxic stress and the upregulation of the UPR^mt^ transcriptional regulators ATF5 and CHOP (Anderson et al., 2019). A multi-OMICS study of CHCHD10 variants linked to ALS demonstrated metabolic disturbances and UPR^mt^ activation (Straub et al., 2021). However, the connection between mutant SOD1, TDP-43, and CHCHD10 needs further investigation.

Studies of ALS patients and animal models indicate altered mitochondrial dynamics in ALS disease pathogenesis. Mitochondrial fragmentation has been observed in ALS models expressing mutant SOD1, potentially due to the downregulation of mitofusins and OPA1 and upregulation of DRP1 and Fis1 in the mouse spinal cord and skeletal muscles (Luo et al., 2013). Altered mitochondrial transport is also evident in ALS motor neurons, which precedes neuronal loss (Magrane et al., 2014). Abnormal mitochondrial transport has also been observed in mutant SOD1 transgenic mice; the mutant SOD1 reduced Miro levels and directly interacted with dynein-dynactin complexes (Shi et al., 2010; Smith et al., 2019). The use of *in vitro* and *in vivo* TDP-43 models has shown a tendency for mitochondria to fragment (Wang W.Z. et al., 2013). In addition, increased DRP1 phosphorylation and decreased OPA1 expression have been reported in mutant TDP-43 transgenic mice (Wang W.Z. et al., 2013). Moreover, several independent studies demonstrated that TDP-43 decreased the expression of mitofusins in patient muscles, transgenic mice, and *Drosophila* neurons by binding to *MFN1* and *MFN2* mRNA (Khalil et al., 2017). Mitochondrial fragmentation has also been reported in neurons and *Drosophila* models expressing WT or mutant FUS (Deng et al., 2015; Chen et al., 2016). Furthermore, shortened mitochondria have been observed in fibroblasts derived from ALS patients expressing mutant CHCHD10 or mutant C9orf72 (Genin et al., 2016; Onesto et al., 2016).

Mitophagy is the most affected MQC mechanism in ALS. Mutations in mitophagy regulators (e.g., VCP, TBK1, and OPTN) are directly linked to ALS (Maruyama et al., 2010; Johnson et al., 2011; Khalil and Lievens, 2017; Oakes et al., 2017). Mutant VCP cannot migrate to impaired mitochondria or recognize ubiquitinated proteins, resulting in the accumulation of damaged mitochondria (Yamano et al., 2016). OPTN and TBK1 mutations interfere with LC3 recruitment to depolarized mitochondria (Li et al., 2016; Ryan and Tumbarello, 2018; Harding et al., 2021). Autophagosomes accumulate in mouse motor neurons expressing mutant SOD1 and patient fibroblasts expressing mutant C9orf72 (Rudnick et al., 2017; Leskela et al., 2021). Mutant SOD1 also suppresses endogenous Miro levels through a Parkin-dependent pathway, resulting in impaired mitochondrial transport and mitophagy (Moller et al., 2017). In the G93A SOD1 mouse model of ALS, decreased MFN2 expression causes defective transport of mitochondria and the calpastatin protein (Wang et al., 2018). In addition, TDP-43 overexpression can cause abnormal aggregation of mitochondria in ALS mouse models (Xu et al., 2010). Furthermore, PINK1 and Parkin protein levels are increased in FUS-overexpressing HEK293 cells (Chen et al., 2016). Conversely, PINK1 or Parkin downregulation reduces abnormal phenotypes in FUS-expressing *Drosophila* (Chen et al., 2016).

### Targeting Mitochondrial Quality Control Rescues Amyotrophic Lateral Sclerosis-Associated Pathology

Finding a cure for ALS has so far been unsuccessful. Previous studies have suggested that modulating MQC is a potential treatment option for ALS. However, treatments to improve mitochondrial function by reducing oxidative stress and apoptosis (e.g., CoQ10, olesoxime, and nortriptyline) have yielded disappointing results in clinical trials despite promising animal studies (Bordet et al., 2007; Wang et al., 2007; Kaufmann et al., 2009). Thus, it is important to determine which types of mitochondrial dysfunction are relevant to this disease and its progression in order to identify new molecular targets for the development of ALS therapies. Sustained treatment of G93A SOD1 transgenic mice with small peptide P110 rescued mitochondrial morphology and improved motor performance and survival (Joshi et al., 2018b). Protein phosphatase 1 (PP1) dephosphorylates DRP1; its suppression prevents mitochondrial fragmentation and ALS-related neuronal damage in primary mutant SOD1 neuronal cultures and iPSC-derived motor neurons (Choi et al., 2020). Inhibiting mitochondrial fission with a dominant-negative DRP1 K38A mutant construct precludes motor neuronal death in mutant SOD1-expressing ALS models (Song et al., 2013). Moreover, induction of mitochondrial fusion by MFN2 overexpression alleviates ALS-TDP-43-induced mitochondrial dysfunction and neuronal damage in spinal cord motor neurons (Wang W. et al., 2013). Miro overexpression also sufficiently rescues mitochondrial axonal transport defects in mutant SOD1 cortical and motor neurons (Moller et al., 2017). Promoting mitochondrial biogenesis with resveratrol or PGC-1α upregulation alleviates the ALS-related syndromes and extends the lifespan of SOD1 G93A and SOD1 G37R transgenic mice, respectively (Markert et al., 2010; Da Cruz et al., 2012). These findings collectively indicate the therapeutic potential of altering mitochondrial dynamics in ALS. Though some data have shown a connection between UPR^mt^ alteration or mitophagy impairment and ALS pathogenesis, whether modulating these MQC pathways protects motor neurons remains to be elucidated.

## Conclusion and Future Perspectives

Maintenance of neuronal homeostasis relies heavily on functional mitochondria, which is highlighted by the fact that mitochondrial dysfunction is often associated with NDs (e.g., AD, PD, HD, and ALS). In addition to producing ATP, mitochondria are home to multiple metabolic processes. Critical strategies exist to regulate mitochondrial integrity. Mitochondrial protein homeostasis is maintained by local chaperones and proteases. The GTPase superfamily proteins regulate mitochondrial morphology, and damaged mitochondria are removed by macroautophagy. These quality control mechanisms co-exist to detect and repair defects that affect mitochondrial function to maintain cellular physiology. Thus, MQC impairment leads to the accumulation of damaged mitochondria, excessive ROS production, energy deficits, and synaptic and neuronal degeneration. There are still many questions regarding the crosstalk between these different MQC mechanisms and their coordination in mitochondrial homeostasis and neurodegenerative disease. For example, the emerging role of UPR^mt^ supports the concept of mitochondrial protein homeostasis, and more importantly, provides a potential mechanism to explain mitochondrial dysfunction observed in neurodegenerative diseases. However, although ATF5 appears to be a functional ortholog of ATFS-1 in *C. elegans*, there is much diversity in mammalian UPR^mt^. Two recent publications suggest that the cleavage of mitochondrial protein DELE1 (DAP3 binding cell death enhancer 1) by inner membrane protease OMA1 is a pathway to transduce the signal of a mitochondrial defect to the cytosol. After being released into the cytosol, the cleaved DELE1 binds to and activates the heme-regulated eIF2α kinase, which phosphorylates eIF2-α and induces the translation of transcription factors ATF4 and CHOP (Fessler et al., 2020; Guo et al., 2020). Therefore, additional transcription factors and proteases other than the canonical ATFS-1/ATF5 signaling pathway can respond to mitochondrial proteo-stress. These findings implicate the coordination of multiple molecular pathways in maintaining mitochondrial proteostasis in mammalian systems. Therefore, previous conclusions on the pathogenic relevance of UPR^mt^ disturbances in neurodegenerative diseases in *C. elegans* should be further validated in mammalian systems. Establishing the mechanism by which UPRmt is impaired during neurodegeneration requires further investigation to identify potential molecular targets for treating NDs.

Despite substantial evidence of the therapeutic advances of targeting MQC in AD, PD, HD, and ALS, any critical discrepancies between experiment models and human subjects should be considered carefully. To date, there are no *in vivo* models recapitulating all the pathological features and disease progression observed in PD patients (Potashkin et al., 2010; Jagmag et al., 2015). Thus, approaches manipulating MQC in rodent or fly models of PD may not be efficacious in patients. In addition, although mitochondrial dysfunction is a common feature shared by different PD models, the underlying pathological mechanism leading to the impairment of MQC is somehow distinguishable among the different models. For example, while mitophagy is mainly affected in mutant PINK1/Parkin models, multiple MQC mechanisms are impaired in α-Syn A53T mutant-related PD models (Martin et al., 2006; Li et al., 2013; Pickrell and Youle, 2015). Therefore, mitophagy modulators identified using PINK1/Parkin models might only partially rescue mitochondrial function or maintain neuronal viability in other PD models. Thus, the therapeutic potential of novel small molecules or peptides targeting MQC should be evaluated in multiple familial and sporadic PD models. Similarly, treatments targeting MQC in AD, HD, and ALS should be validated in different models.

Data from several recent studies indicate that MQC mechanisms seem to regulate each other. Haeussler et al. (2020) found that UPR^mt^ activation can stimulate mitochondrial fission, and reversely, whereas blocking mitochondrial fusion can induce the UPR^mt^ response under physiological conditions in *C. elegans*. These data suggest that mitochondrial fission and UPR^mt^ may be activated simultaneously, providing a cue for the concomitant activation of UPR^mt^ and mitochondrial fragmentation in NDs. Nevertheless, because of such mutual regulation of UPR^mt^ and mitochondrial dynamics, therapeutic approaches seeking to upregulate UPR^mt^ in NDs should be considered carefully since excessive fission would impair mitochondrial function. Moreover, mitochondrial proteolytic stress can be rescued by Parkin and PINK1-mediated mitophagy (Jin and Youle, 2013; Burbulla et al., 2014), suggesting that mitophagy may alleviate mitochondrial proteotoxicity. It is clear that mitophagy functions to eliminate damaged mitochondria. Therefore the upregulation of mitophagy could enrich the pool of healthy mitochondria in NDs, such as PD, where there is an accumulation of stressed and damaged mitochondria. However, a recent study showed that the activation of mitophagy inhibited UPR^mt^ activation in *C. elegans* (Haeussler et al., 2020). Unlike the mitophagy that targets mitochondria with irreversible damage, UPR^mt^ activation affects the function of the overall mitochondrial pool in the cell as a consequence of transcriptional regulation. Given that UPR^mt^ activation induces genes that promote mitochondrial biogenesis and functions, it is necessary to evaluate the side effects of therapeutic strategies aiming to upregulate mitophagy on the mitochondrial function within the healthy pool. Therefore, understanding the connection between these MQCs could provide cues for developing efficient and safe treatments for NDs.

Whether a combination treatment targeting multiple pathways may provide a better therapeutic effect against NDs needs further investigation. Although improving mitochondrial function by targeting MQC can prevent or slow disease progression *in vivo*, it remains unclear whether these modulations can reverse the volume of neurons in the CNS. Further investigation is also required to understand the side effects and administration methods of targeting MQC for neurodegenerative treatments. Our current knowledge of MQC continues to evolve, providing a novel research scheme for developing practical therapeutic approaches to combat NDs.

## Author Contributions

DH made substantial contribution to the conception and design of the study. DH and ZL participated in drafting the manuscript. XQ revised the manuscript. All authors contributed to the article and approved the submitted version.

## Conflict of Interest

The authors declare that the research was conducted in the absence of any commercial or financial relationships that could be construed as a potential conflict of interest.

## Publisher’s Note

All claims expressed in this article are solely those of the authors and do not necessarily represent those of their affiliated organizations, or those of the publisher, the editors and the reviewers. Any product that may be evaluated in this article, or claim that may be made by its manufacturer, is not guaranteed or endorsed by the publisher.
